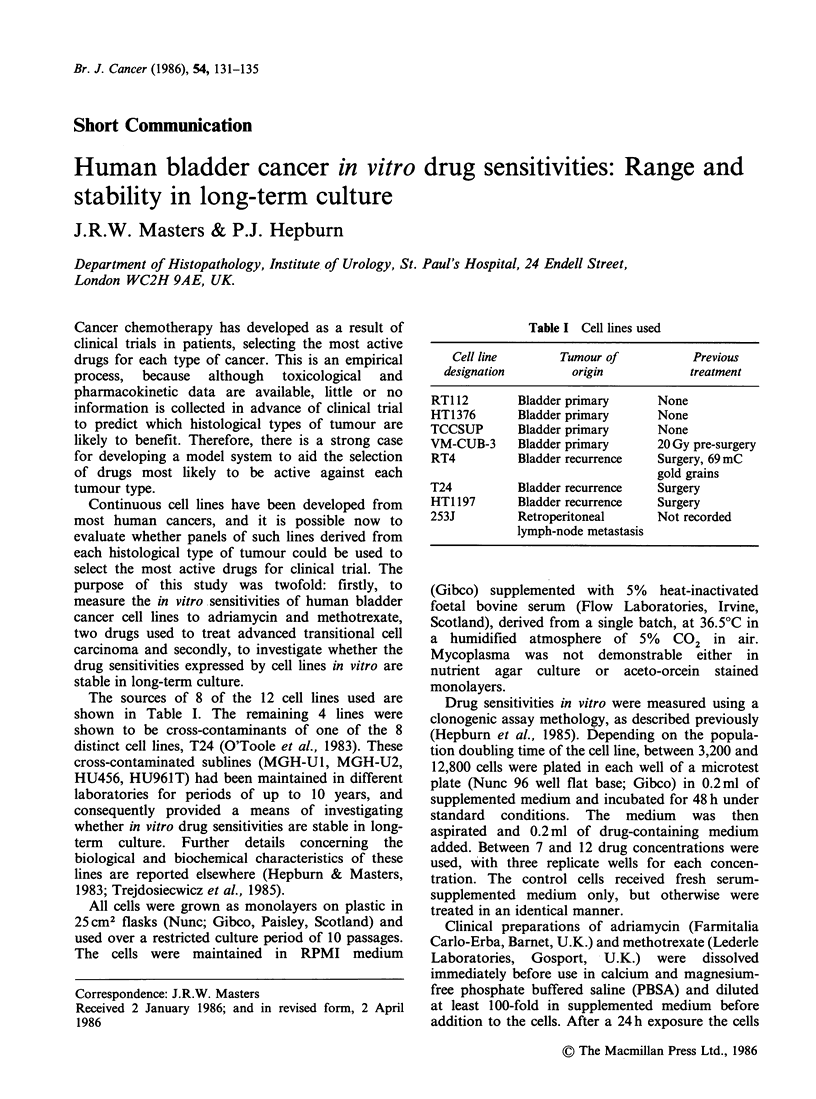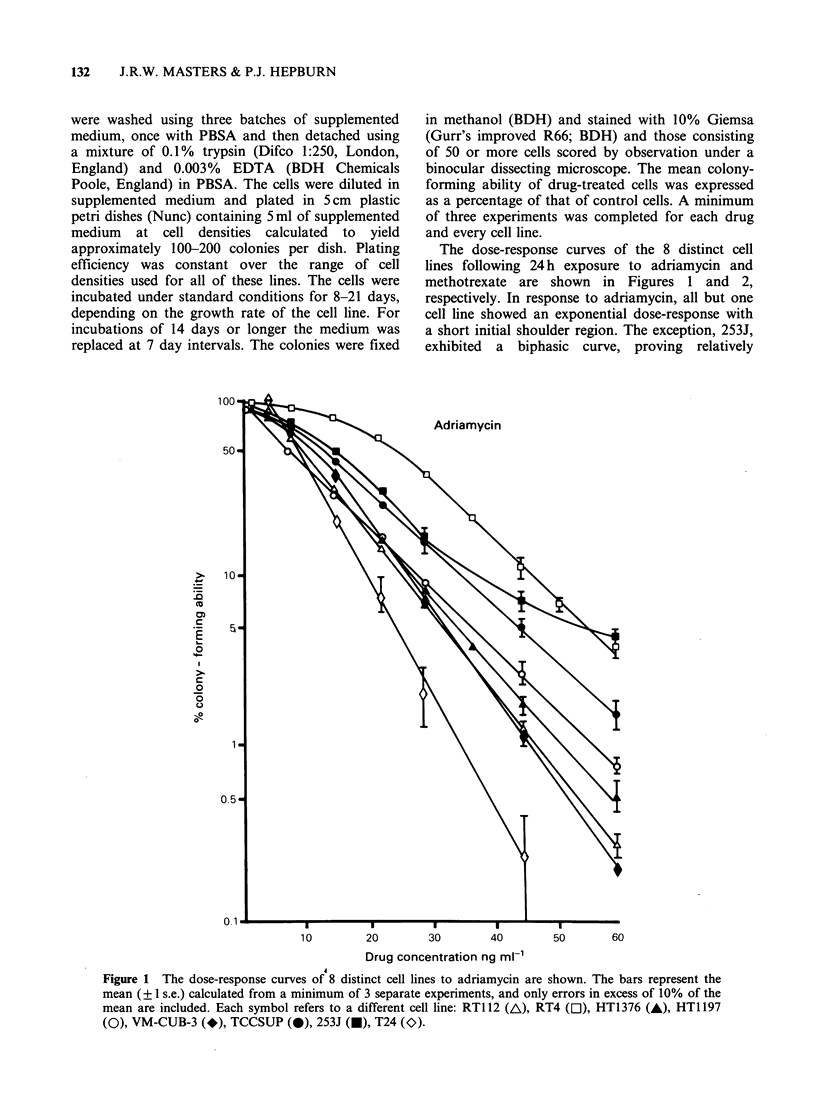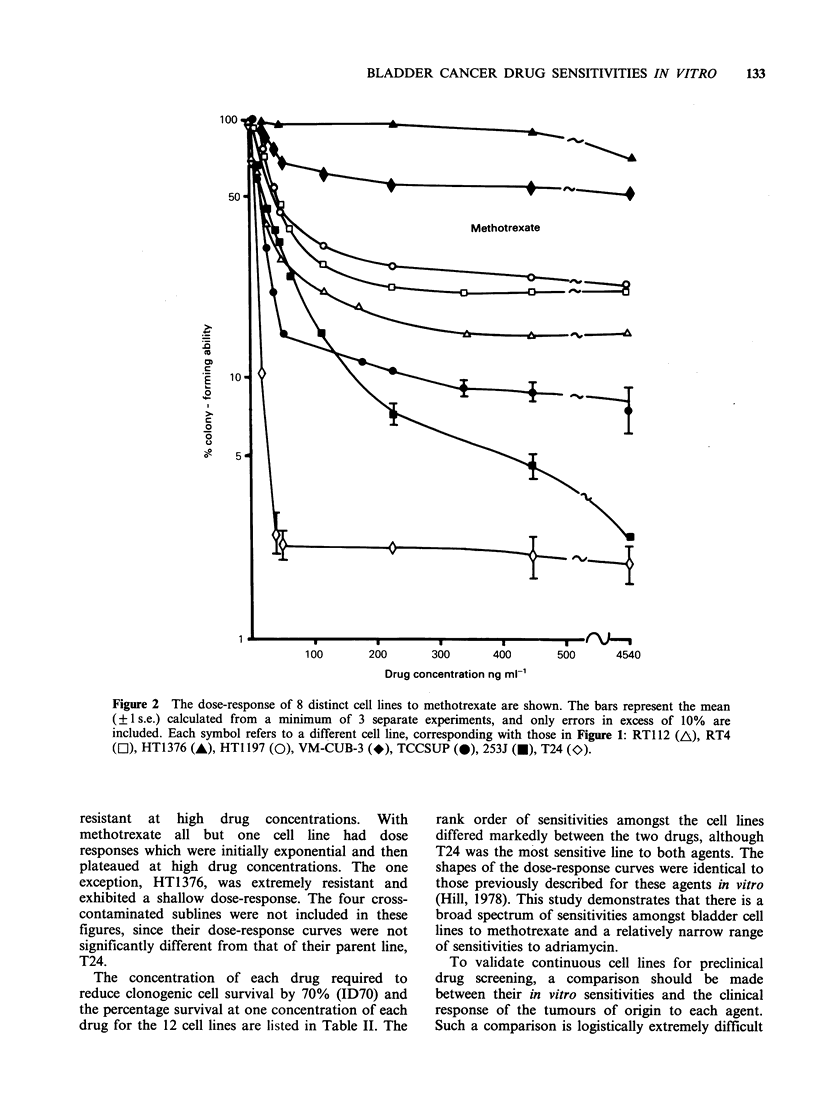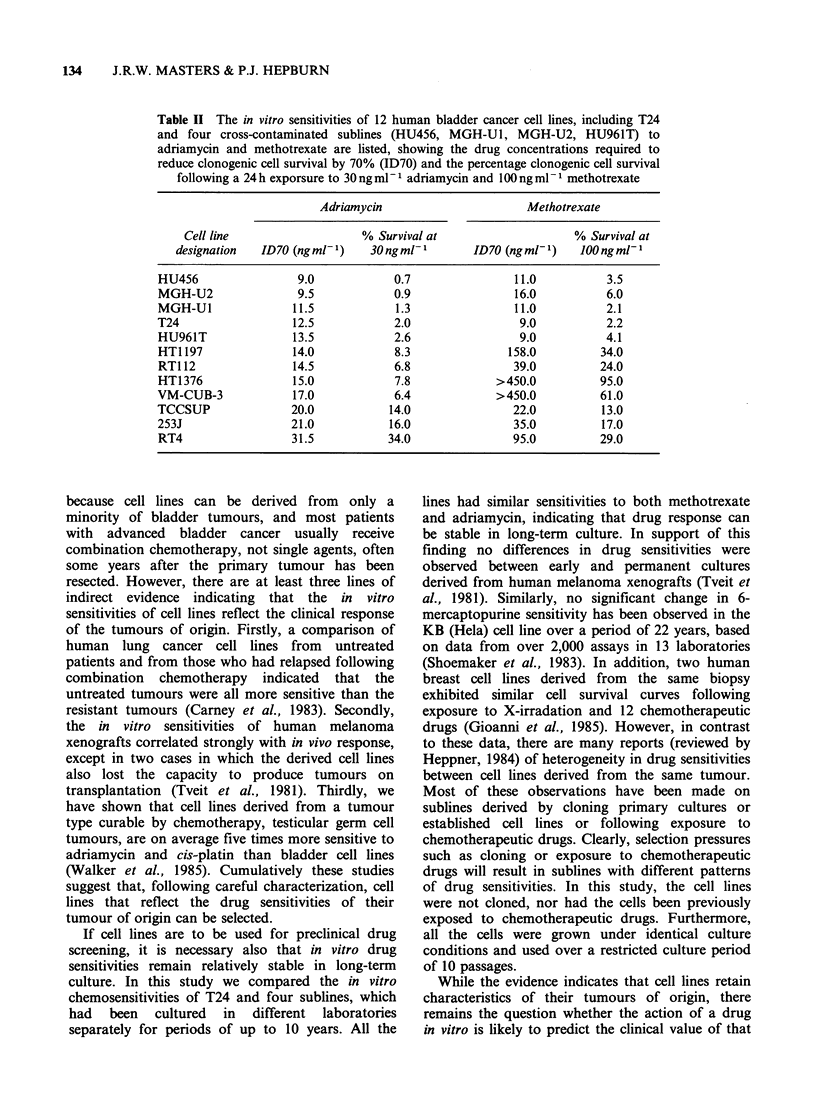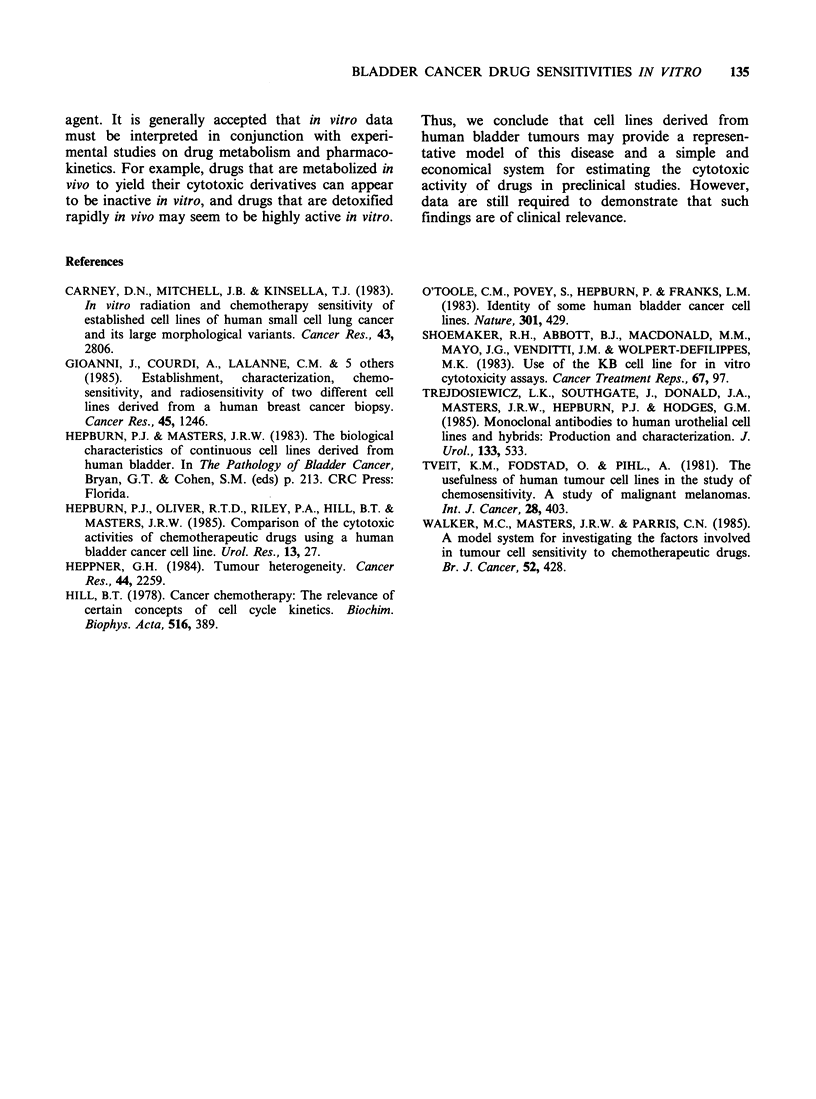# Human bladder cancer in vitro drug sensitivities: range and stability in long-term culture.

**DOI:** 10.1038/bjc.1986.163

**Published:** 1986-07

**Authors:** J. R. Masters, P. J. Hepburn


*Br. J. Cancer* (1986), **54**, 131–135

**Short Communication**

### Human bladder cancer *in vitro* drug sensitivities: Range and stability in long-term culture

J.R.W. Masters & P.J. Hepburn

*Department of Histopathology, Institute of Urology, St. Paul's Hospital, 24 Endell Street, London WC2H 9AE, UK.*

Cancer chemotherapy has developed as a result of clinical trials in patients, selecting the most active drugs for each type of cancer. This is an empirical process, because although toxicological and pharmacokinetic data are available, little or no information is collected in advance of clinical trial to predict which histological types of tumour are likely to benefit. Therefore, there is a strong case for developing a model system to aid the selection of drugs most likely to be active against each tumour type.

Continuous cell lines have been developed from most human cancers, and it is possible now to evaluate whether panels of such lines derived from each histological type of tumour could be used to select the most active drugs for clinical trial. The purpose of this study was twofold: firstly, to measure the *in vitro* sensitivities of human bladder cancer cell lines to adriamycin and methotrexate, two drugs used to treat advanced transitional cell carcinoma and secondly, to investigate whether the drug sensitivities expressed by cell lines *in vitro* are stable in long-term culture.

The sources of 8 of the 12 cell lines used are shown in Table I. The remaining 4 lines were shown to be cross-contaminants of one of the 8 distinct cell lines, T24 (O'Toole *et al.*, 1983). These cross-contaminated sublines (MGH-U1, MGH-U2, HU456, HU961T) had been maintained in different laboratories for periods of up to 10 years, and consequently provided a means of investigating whether *in vitro* drug sensitivities are stable in long-term culture. Further details concerning the biological and biochemical characteristics of these lines are reported elsewhere (Hepburn & Masters, 1983; Trejdosiecwicz *et al.*, 1985).

All cells were grown as monolayers on plastic in 25 cm² flasks (Nunc; Gibco, Paisley, Scotland) and used over a restricted culture period of 10 passages. The cells were maintained in RPMI medium (Gibco) supplemented with 5% heat-inactivated foetal bovine serum (Flow Laboratories, Irvine, Scotland), derived from a single batch, at 36.5°C in a humidified atmosphere of 5% $CO_2$ in air. Mycoplasma was not demonstrable either in nutrient agar culture or aceto-orcein stained monolayers.

**Table I** Cell lines used

| *Cell line designation* | *Tumour of origin* | *Previous treatment* |
|---|---|---|
| RT112 | Bladder primary | None |
| HT1376 | Bladder primary | None |
| TCCSUP | Bladder primary | None |
| VM-CUB-3 | Bladder primary | 20 Gy pre-surgery |
| RT4 | Bladder recurrence | Surgery, 69 mC gold grains |
| T24 | Bladder recurrence | Surgery |
| HT1197 | Bladder recurrence | Surgery |
| 253J | Retroperitoneal lymph-node metastasis | Not recorded |

Drug sensitivities *in vitro* were measured using a clonogenic assay methology, as described previously (Hepburn *et al.*, 1985). Depending on the population doubling time of the cell line, between 3,200 and 12,800 cells were plated in each well of a microtest plate (Nunc 96 well flat base; Gibco) in 0.2 ml of supplemented medium and incubated for 48 h under standard conditions. The medium was then aspirated and 0.2 ml of drug-containing medium added. Between 7 and 12 drug concentrations were used, with three replicate wells for each concentration. The control cells received fresh serum-supplemented medium only, but otherwise were treated in an identical manner.

Clinical preparations of adriamycin (Farmitalia Carlo-Erba, Barnet, U.K.) and methotrexate (Lederle Laboratories, Gosport, U.K.) were dissolved immediately before use in calcium and magnesium-free phosphate buffered saline (PBSA) and diluted at least 100-fold in supplemented medium before addition to the cells. After a 24 h exposure the cells

Correspondence: J.R.W. Masters
Received 2 January 1986; and in revised form, 2 April 1986

© The Macmillan Press Ltd., 1986

were washed using three batches of supplemented medium, once with PBSA and then detached using a mixture of 0.1% trypsin (Difco 1:250, London, England) and 0.003% EDTA (BDH Chemicals Poole, England) in PBSA. The cells were diluted in supplemented medium and plated in 5 cm plastic petri dishes (Nunc) containing 5 ml of supplemented medium at cell densities calculated to yield approximately 100–200 colonies per dish. Plating efficiency was constant over the range of cell densities used for all of these lines. The cells were incubated under standard conditions for 8–21 days, depending on the growth rate of the cell line. For incubations of 14 days or longer the medium was replaced at 7 day intervals. The colonies were fixed in methanol (BDH) and stained with 10% Giemsa (Gurr's improved R66; BDH) and those consisting of 50 or more cells scored by observation under a binocular dissecting microscope. The mean colony-forming ability of drug-treated cells was expressed as a percentage of that of control cells. A minimum of three experiments was completed for each drug and every cell line.

The dose-response curves of the 8 distinct cell lines following 24 h exposure to adriamycin and methotrexate are shown in Figures 1 and 2, respectively. In response to adriamycin, all but one cell line showed an exponential dose-response with a short initial shoulder region. The exception, 253J, exhibited a biphasic curve, proving relatively

**Figure 1** The dose-response curves of 8 distinct cell lines to adriamycin are shown. The bars represent the mean (±1 s.e.) calculated from a minimum of 3 separate experiments, and only errors in excess of 10% of the mean are included. Each symbol refers to a different cell line: RT112 (△), RT4 (□), HT1376 (▲), HT1197 (○), VM-CUB-3 (◆), TCCSUP (●), 253J (■), T24 (◇).

**Figure 2** The dose-response of 8 distinct cell lines to methotrexate are shown. The bars represent the mean (±1 s.e.) calculated from a minimum of 3 separate experiments, and only errors in excess of 10% are included. Each symbol refers to a different cell line, corresponding with those in **Figure 1**: RT112 (△), RT4 (□), HT1376 (▲), HT1197 (○), VM-CUB-3 (◆), TCCSUP (●), 253J (■), T24 (◇).

resistant at high drug concentrations. With methotrexate all but one cell line had dose responses which were initially exponential and then plateaued at high drug concentrations. The one exception, HT1376, was extremely resistant and exhibited a shallow dose-response. The four cross-contaminated sublines were not included in these figures, since their dose-response curves were not significantly different from that of their parent line, T24.

The concentration of each drug required to reduce clonogenic cell survival by 70% (ID70) and the percentage survival at one concentration of each drug for the 12 cell lines are listed in Table II. The rank order of sensitivities amongst the cell lines differed markedly between the two drugs, although T24 was the most sensitive line to both agents. The shapes of the dose-response curves were identical to those previously described for these agents *in vitro* (Hill, 1978). This study demonstrates that there is a broad spectrum of sensitivities amongst bladder cell lines to methotrexate and a relatively narrow range of sensitivities to adriamycin.

To validate continuous cell lines for preclinical drug screening, a comparison should be made between their *in vitro* sensitivities and the clinical response of the tumours of origin to each agent. Such a comparison is logistically extremely difficult

**Table II** The *in vitro* sensitivities of 12 human bladder cancer cell lines, including T24 and four cross-contaminated sublines (HU456, MGH-U1, MGH-U2, HU961T) to adriamycin and methotrexate are listed, showing the drug concentrations required to reduce clonogenic cell survival by 70% (ID70) and the percentage clonogenic cell survival following a 24 h exporsure to 30 ng ml$^{-1}$ adriamycin and 100 ng ml$^{-1}$ methotrexate

| | *Adriamycin* | | *Methotrexate* | |
|---|---|---|---|---|
| *Cell line designation* | *ID70 (ng ml$^{-1}$)* | *% Survival at 30 ng ml$^{-1}$* | *ID70 (ng ml$^{-1}$)* | *% Survival at 100 ng ml$^{-1}$* |
| HU456 | 9.0 | 0.7 | 11.0 | 3.5 |
| MGH-U2 | 9.5 | 0.9 | 16.0 | 6.0 |
| MGH-U1 | 11.5 | 1.3 | 11.0 | 2.1 |
| T24 | 12.5 | 2.0 | 9.0 | 2.2 |
| HU961T | 13.5 | 2.6 | 9.0 | 4.1 |
| HT1197 | 14.0 | 8.3 | 158.0 | 34.0 |
| RT112 | 14.5 | 6.8 | 39.0 | 24.0 |
| HT1376 | 15.0 | 7.8 | >450.0 | 95.0 |
| VM-CUB-3 | 17.0 | 6.4 | >450.0 | 61.0 |
| TCCSUP | 20.0 | 14.0 | 22.0 | 13.0 |
| 253J | 21.0 | 16.0 | 35.0 | 17.0 |
| RT4 | 31.5 | 34.0 | 95.0 | 29.0 |

because cell lines can be derived from only a minority of bladder tumours, and most patients with advanced bladder cancer usually receive combination chemotherapy, not single agents, often some years after the primary tumour has been resected. However, there are at least three lines of indirect evidence indicating that the *in vitro* sensitivities of cell lines reflect the clinical response of the tumours of origin. Firstly, a comparison of human lung cancer cell lines from untreated patients and from those who had relapsed following combination chemotherapy indicated that the untreated tumours were all more sensitive than the resistant tumours (Carney *et al.*, 1983). Secondly, the *in vitro* sensitivities of human melanoma xenografts correlated strongly with *in vivo* response, except in two cases in which the derived cell lines also lost the capacity to produce tumours on transplantation (Tveit *et al.*, 1981). Thirdly, we have shown that cell lines derived from a tumour type curable by chemotherapy, testicular germ cell tumours, are on average five times more sensitive to adriamycin and *cis*-platin than bladder cell lines (Walker *et al.*, 1985). Cumulatively these studies suggest that, following careful characterization, cell lines that reflect the drug sensitivities of their tumour of origin can be selected.

If cell lines are to be used for preclinical drug screening, it is necessary also that *in vitro* drug sensitivities remain relatively stable in long-term culture. In this study we compared the *in vitro* chemosensitivities of T24 and four sublines, which had been cultured in different laboratories separately for periods of up to 10 years. All the lines had similar sensitivities to both methotrexate and adriamycin, indicating that drug response can be stable in long-term culture. In support of this finding no differences in drug sensitivities were observed between early and permanent cultures derived from human melanoma xenografts (Tveit *et al.*, 1981). Similarly, no significant change in 6-mercaptopurine sensitivity has been observed in the KB (Hela) cell line over a period of 22 years, based on data from over 2,000 assays in 13 laboratories (Shoemaker *et al.*, 1983). In addition, two human breast cell lines derived from the same biopsy exhibited similar cell survival curves following exposure to X-irradation and 12 chemotherapeutic drugs (Gioanni *et al.*, 1985). However, in contrast to these data, there are many reports (reviewed by Heppner, 1984) of heterogeneity in drug sensitivities between cell lines derived from the same tumour. Most of these observations have been made on sublines derived by cloning primary cultures or established cell lines or following exposure to chemotherapeutic drugs. Clearly, selection pressures such as cloning or exposure to chemotherapeutic drugs will result in sublines with different patterns of drug sensitivities. In this study, the cell lines were not cloned, nor had the cells been previously exposed to chemotherapeutic drugs. Furthermore, all the cells were grown under identical culture conditions and used over a restricted culture period of 10 passages.

While the evidence indicates that cell lines retain characteristics of their tumours of origin, there remains the question whether the action of a drug *in vitro* is likely to predict the clinical value of that agent. It is generally accepted that *in vitro* data must be interpreted in conjunction with experimental studies on drug metabolism and pharmacokinetics. For example, drugs that are metabolized *in vivo* to yield their cytotoxic derivatives can appear to be inactive *in vitro*, and drugs that are detoxified rapidly *in vivo* may seem to be highly active *in vitro*.

Thus, we conclude that cell lines derived from human bladder tumours may provide a representative model of this disease and a simple and economical system for estimating the cytotoxic activity of drugs in preclinical studies. However, data are still required to demonstrate that such findings are of clinical relevance.

**References**

CARNEY, D.N., MITCHELL, J.B. & KINSELLA, T.J. (1983). *In vitro* radiation and chemotherapy sensitivity of established cell lines of human small cell lung cancer and its large morphological variants. *Cancer Res.*, **43**, 2806.

GIOANNI, J., COURDI, A., LALANNE, C.M. & 5 others (1985). Establishment, characterization, chemosensitivity, and radiosensitivity of two different cell lines derived from a human breast cancer biopsy. *Cancer Res.*, **45**, 1246.

HEPBURN, P.J. & MASTERS, J.R.W. (1983). The biological characteristics of continuous cell lines derived from human bladder. In *The Pathology of Bladder Cancer*, Bryan, G.T. & Cohen, S.M. (eds) p. 213. CRC Press: Florida.

HEPBURN, P.J., OLIVER, R.T.D., RILEY, P.A., HILL, B.T. & MASTERS, J.R.W. (1985). Comparison of the cytotoxic activities of chemotherapeutic drugs using a human bladder cancer cell line. *Urol. Res.*, **13**, 27.

HEPPNER, G.H. (1984). Tumour heterogeneity. *Cancer Res.*, **44**, 2259.

HILL, B.T. (1978). Cancer chemotherapy: The relevance of certain concepts of cell cycle kinetics. *Biochim. Biophys. Acta*, **516**, 389.

O'TOOLE, C.M., POVEY, S., HEPBURN, P. & FRANKS, L.M. (1983). Identity of some human bladder cancer cell lines. *Nature*, **301**, 429.

SHOEMAKER, R.H., ABBOTT, B.J., MACDONALD, M.M., MAYO, J.G., VENDITTI, J.M. & WOLPERT-DEFILIPPES, M.K. (1983). Use of the KB cell line for in vitro cytotoxicity assays. *Cancer Treatment Reps.*, **67**, 97.

TREJDOSIEWICZ, L.K., SOUTHGATE, J., DONALD, J.A., MASTERS, J.R.W., HEPBURN, P.J. & HODGES, G.M. (1985). Monoclonal antibodies to human urothelial cell lines and hybrids: Production and characterization. *J. Urol.*, **133**, 533.

TVEIT, K.M., FODSTAD, O. & PIHL., A. (1981). The usefulness of human tumour cell lines in the study of chemosensitivity. A study of malignant melanomas. *Int. J. Cancer*, **28**, 403.

WALKER, M.C., MASTERS, J.R.W. & PARRIS, C.N. (1985). A model system for investigating the factors involved in tumour cell sensitivity to chemotherapeutic drugs. *Br. J. Cancer*, **52**, 428.